# Typology and characteristics of indigenous goats and production systems in different agro-ecological zones of Tanzania

**DOI:** 10.1007/s11250-022-03074-1

**Published:** 2022-01-19

**Authors:** Athumani Nguluma, Martina Kyallo, Getinet Mekuriaw Tarekegn, Rose Loina, Zabron Nziku, Sebastian Chenyambuga, Roger Pelle

**Affiliations:** 1 Tanzania Livestock Research Institute (TALIRI), P. O. Box 834, Dodoma, Tanzania; 2 Sokoine University of Agriculture, P. O Box 3000, Morogoro, Tanzania; 3 Biosciences Eastern and Central Africa-International Livestock Research Institute (BecA-ILRI) Hub, Nairobi, Kenya; 4 Department of Animal Breeding and Genetics, Swedish University of Agricultural Sciences (SLU), Uppsala, Sweden; 5 Department of Animal Production and Technology, Bahir Dar University, Bahir Dar, Ethiopia

**Keywords:** Phenotypic characterization, Production system, Coat color, Cluster analysis

## Abstract

Tanzania has a goat population of about 24.8 million most of which belong to the Small East African breed distributed in almost all agro-ecological zones. The different goat populations and the production system in which they are raised are not well characterized depriving animal breeders useful information in designing and running improvement and conservation programs. Therefore, the study was conducted in all agro-ecological zones in Tanzania to characterize the indigenous goats and the production system in which they are raised. Data on animals were collected from 688 randomly selected adult female goats and for production system description; 220 households were interviewed. Analysis of variance and discriminant analysis were used on quantitative data, while frequency analysis was used on qualitative data. Income generation and meat production were the primary goat rearing objectives. More than 55% of respondents grazed their animals freely in communal lands where natural pasture was the chief feed resource. Mating was mainly uncontrolled with apron and castration being used by goat keepers as mating control methods. Common diseases were contagious caprine pleural pneumonia and helminthiasis. Feed shortage, prevalence of diseases, and water scarcity were the major goat production constraints. There were morphological variations between and within these goat populations, and based on quantitative data, the goats were categorized into two groups. High twinning was observed in Ujiji and Lindi goats and low for Sukuma. The dominant coat color was plain white in Pare, Gogo, Maasai, and Tanga. Other coat color patterns were mixed black and white for Sukuma, reddish-brown for Lindi, black and reddish-brown for Ujiji, and white and reddish-brown for Pwani and Maasai. High within population variation is observed which is important as it can be used as a basis for genetic improvement through selection.

## Introduction

Tanzania has a goat population of 24.8 million (NBS 2020) most of which belong to the Small East African (SEA) breed distributed in almost all agro-ecological zones. The animals are important for the livelihood of the resource-poor farmers especially in rural areas as they contribute towards income generation, meat and milk production for the family, and other socio-cultural functions. The SEA goats are regarded as hardy and comparatively outperform the upgraded in many farmers’ valued traits such as disease and survivability. However, due to long kidding interval of 12 months (NEI 1999), advanced age at first kidding of 18–24 months (MAFS 2002), small mature size of 24–28 kg (Chenyambuga et al. 2012), small carcass weight of 12 kg (MAFS 2002), and low milk production, the indigenous goats in Tanzania are regarded as low producers compared to their exotic counterparts.

Efforts to improve goat production and productivity in the country have been made using three approaches (Chenyambuga and Lekule 2014) including the introduction of exotic dairy goat breeds from temperate countries to be used directly for production. Through this approach, dairy goat breeds such as Saanen, Alpine, Anglo-Nubian, and Toggenburg were introduced in the country in the early 1960s (Das and Sendalo 1991) and were used mainly in high potential highland areas where the environment was conducive for them to survive and produce. Another approach involved crossbreeding of the SEA does with exotic bucks for meat production, whereby Boer bucks were introduced and crossed with indigenous does to produce F1 crossbreds that grew faster and had higher mature weight than the pure local ones. The third approach was the development of the synthetic breed in which a three-way dual-purpose goat breed, known as “Blended” goat was developed from the crossing of SEA, Boer, and Kamorai goats (Das and Sendalo 1991). However, the three approaches have not been successful due to lack of adaptability and resistance to various diseases and parasites of the pure exotic and their crossbreds and high cost involved in maintaining them which is not affordable to smallholder farmers.

Selection within the local populations is proposed as a sustainable strategy for the improvement of the local animals in developing countries (Syrstad and Ruane, 1998) as it can sustain local breeds and secure conservation of the genetic resources. Breed-specific information is required before embarking on conservation and improvement through selection. Although various studies have been done previously to characterize the indigenous goats, they have largely focused on few populations and agro-ecologies and thus conceded numerous shortcomings when a collective comparison of all the agro-ecological zones (AEZs) and populations is needed. Therefore, this study was carried out in order to characterize all the indigenous goat populations in Tanzania and the production system in which they are raised.

## Material and methods

### Study areas and sampling strategy

Tanzania with a total land area of 945,087 km^2^ is located on the eastern coast of Africa, south of the equator between latitudes 1^0^00' and 11^0^48'S and longitudes 29^0^30' and 39^0^45’E. Tanzania has seven main AEZs varying in altitude, rainfall patterns, soil types, and physiographic features, (Malozo 2014) although there are numerous smaller ones (Table 1). A hierarchical sampling procedure was followed where the big sampling frames were agro-ecological zones. A rapid assessment of the available published and unpublished data and discussion with the regional livestock experts was made before the main data collection to know the distribution of the targeted goat populations in each study area. Based on the information from the secondary data and information available in the regional extension offices, districts that were representative and had indigenous goat production potential were purposively selected. During selection of the districts, the distribution and density of the respective goat populations and accessibility were considered. A total of 220 households (a minimum of 17 households for each goat population) were selected for interview from the households who owned at least three unrelated adult female goats that had given birth at least once using a systematic random sampling procedure.

**Table 1:** Agro-ecological zones of Tanzania and the sampled populations

AEZ	Subzone	Sampled population	Altitude	Rainfall
Coast	North: Tanga, Coast, and Dar es Salaam South: eastern Lindi and Mtwara	Tanga, Lindi, Pwani and Newala	Under 300 m	North: bimodal, 750–1200 mm South: unimodal, 800–1200 mm
Arid	North: Serengeti, Tarangire, and Ngorongoro parks, part of Masailand, Masai steppe, Mkomazi reserve, Pangani and eastern Dodoma	Maasai	North: 1300–1800 m South: 500–1500 m	North: bimodal, unreliable, 500–600 mm South: bimodal and unreliable, 400–600 mm
Semi-arid	Dodoma, Singida, Arusha, Shinyanga Southern: Morogoro, Lindi and Southwest Mtwara	Gogo	Central: 1000–1500 m Southeastern: 200–600 m	Central: unimodal and unreliable: 500–800 mm Southeastern: unimodal, 600–800 mm
Plateaux	Western: Tabora, Rukwa, and Mbeya North: Kigoma, Mara,Ruvuma and Morogoro	Sukuma	800–1500 m	Western: unimodal, 800–1000 mm Southern: unimodal, very reliable, 900–1300 mm
Southern and western highlands	Southern: Morogoro, Iringa, and Mbeya, Sumbawanga Western: shore of Lake Tanganyika in Kigoma and Kagera	Songwe, Fipa and Ujiji	Southern: 120–1500 m Western:100–1800 m Southwestern: 1400–2300 m	Southern: unimodal, reliable, 800–1400 mm Southern: unimodal, reliable, 800–1000 mm Western: bimodal, 1000–2000 mm
Nothern highlands	Northern: Kilimanjaro, Meru, and Pare mountains	Pare	Northern: 1000–2500 m Granitic mts: 1000–2000 m	Northern: bimodal, varies widely, 1000–2000 mm Granitic mts: bimodal and very reliable, 1000–2000 m

### Data collection

A checklist and semi-structured questionnaire were administered to the selected respondents by a team of enumerators trained on methods and approaches on phenotypic characterization of animal genetic resources (AnGRs) and who spoke Swahili (the national language) and where necessary the questions were translated to the language of the respondents. Information on household socioeconomic characteristics, socio-cultural importance of goats, management practices, breeding system, unique adaptive character, goat feeding and watering, production constraints, and other related issues were collected using semi-structured questionnaires. Secondary information such as temperature, precipitation, agroecology, and livestock and livestock population demography of the study areas was accessed from district and ward extension offices from published and unpublished secondary data sources.

Based on breed morphological characteristics descriptor list of FAO (2012) for morphological characterization of goats, both qualitative and quantitative data were collected from 688 heads of adult (4 pair of permanent incisors) female goats comprising of Gogo (*n* = 73), Lindi (*n* = 54), Maasai (*n* = 82), Newala (*n* = 54), Pare (68), Pwani (55), Fipa (54), Songwe (54), Sukuma (69), Tanga (54), and Ujiji (71). To avoid the genetic similarity of goats, up to 3 animals per household (based on the number of goats) were used for both qualitative and quantitative trait recording. For each goat, five quantitative traits were measured, i.e., body weight (BW), chest girth (CG), height at wither (HW), body length (BL), and rump height (RH). Body weight in kg was measured using a Salter hanging spring-type scale (Salter Housewares, Tonbridge, UK). The linear measurements were taken using a measuring tape (Shanghai Kearing Stationery Co., Ltd., Shanghai, China) after making the animal stand squarely on even ground and recorded in centimeters. Qualitative traits such as coat color pattern, coat color type, horn presence, horn shape, horn orientation, ear orientation, facial (head) profile, wattles presence, and beard presence were recorded.

### Data analysis

Quantitative data were analyzed using Statistical Analysis System Version 9.2 (SAS 2008), whereas qualitative data were analyzed by SPSS Package 20 (SPSS 2020). Basic statistics including mean and standard error were computed for quantitative variables or body measurements and frequency and percentage for qualitative variables. A general linear model (GLM) procedure of SAS and R was used to analyze the quantitative data. Data were analyzed by fitting linear body measurements as dependent variables and goat population as a fixed factor and the magnitudes of quantitative variables were expressed as least square means (± SE). Chi-square test was used to test whether there is a significant difference at a 5% level of significance between the observed frequencies in two or more categories.

The following fixed-effect models were used to analyze morphological body measurements.

$$Yij =\mu + Bi+\varepsilon ij$$

where.

*Yij* = observed quantitative measurement of trait of interest.

*μ* = population mean.

*Bi* = ith goat population effect (*i* = 1, 2, 3).

*εij* = random error associated with quantitative body measurements .

The quantitative variables were subjected to discriminant (DISCRIM) and canonical discriminant analysis (CANDISC) procedure of SAS to ascertain the existence of population-level phenotypic differences among the goat populations. Multivariate discriminant analysis was conducted using quantitative traits to determine the percentage assignment of each individual to their respective populations, to distinguish significant discriminating power of different traits and to obtain distances between populations. Hierarchical cluster analysis was performed using quantitative variables, and dendrogram was constructed based on Euclidean distance between goat populations using unweighted pair-group method to group the goat populations into their morphological similarity.

## Results

### Purpose of keeping goats

Analysis of multiple response questions on the purpose of keeping goats is presented in Table 2. The majority emphasized income generation and meat production as the major purposes of keeping goats. Other reasons such as milk, manure and skin production, breeding, saving, and cultural purposes were mentioned but were given very low emphasis except for Pare goats in same districts where more than half of the farmers mentioned milk production as one of the purposes of keeping goats.

**Table 2:** Percentages of respondents for different purposes of goat keeping in Tanzania

Purpose	Gogo	Lindi	Maasai	Newala	Pare	Pwani	Fipa	Songwe	Sukuma	Tanga	Ujiji
Income	97.5	92.1	90	90.2	92.5	93.4	95.7	92.1	91.2	96.3	97.5
Meat	78.8	84.1	86.3	80.3	95	83.2	90.6	89.4	86.2	94.2	97.5
Milk	10	5.2	53.8	25.7	55	5.3	3.2	6	1.2	3.1	1.8
Manure	30.2	26.7	48.8	4.3	35.6	7.5	35.2	19.1	27.3	12.2	3.6
Breeding	5	7.2	11.3	2.8	14.1	3.3	4.3	2	10.4	2.1	1.4
Saving	12.2	10.2	8.8	15.3	9.4	7.2	13.2	20.2	18.2	21	17.6
Skin	4.1	1.3	2.5	2.1	1.2	1.7	2.3	3.1	21.3	1.2	2.4
Cultural	15.3	7.2	21.3	6.6	25.1	4.3	17.3	15.6	26.7	7.9	31.2

### General goat husbandry practices

Information was sought on different aspects of management of their animals such as feed resources and feeding practices, sources of water, and housing (Table 3). Kraal or boma which is an enclosure fenced with thorn tree branches with no roof was the main housing type used by the majority of respondents. A sizable proportion of farmers also housed their goats with other livestock species, while a few kept them in the houses where they lived. The majority of the respondents mentioned free grazing as the major feeding system used and the main feed resources were natural pastures or shrubs. In some districts, a significant proportion (40–56%) of farmers also reported tethering their animals as one of the feeding systems they used together with free grazing. In most parts, farmers reported practicing supplementation mainly using crop residues. Very few reported use of conventional commercial supplementation except in Songwe and Fipa regions where about 50% of farmers used homemade maize bran. The different sources of water for the animals reported by the farmers were rivers, water pipes, rain, springs, and boreholes depending on availability and seasons of the year. Across the study areas, majority (more than 60%) of the farmers interviewed reported to practice uncontrolled mating, and castration was the main method used in areas where mating control was practiced except among the Maasai goat keepers where the apron was used by 58% of the farmers. In addition, separation of males was done to control mating but to a very low extent except in Tanga region where nearly half (48.3%) reported to separate their bucks during grazing to control mating. The majority of the goat keepers used breeding bucks born within their flocks and a few purchased or used bucks from their neighborhood. Culling of animals was practiced by only a small proportion of farmers and the main culling criteria used were old age, poor fertility, poor health, and poor body condition.

**Table 3:** Percentages of respondents for different goat husbandry practices of the farmers

Practice	Level	Population										
		Gogo	Lindi	Maasai	Newala	Pare	Pwani	Fipa	Songwe	Sukuma	Tanga	Ujiji
Feeding system	Tethering	6.6	33.8	8.5	40.4	10.4	47.2	56.4	43.8	26.6	45.4	30.2
	Free grazing	82.2	61.3	79	70.3	88.5	55.5	60	72.2	74.2	54.9	88.5
	Stall feeding	1.2	26.3	12.5	20.6	1.1	16.3	17.6	10.3	7.2	31.6	10.3
Major feed resources	Natural Pastures/shrubs	95.1	93.2	92.5	90.4	97.3	90.6	97.8	94.9	97.1	88	97.2
	Established forage trees	2.5	4.4	4.3	8.3	1.5	6.3	1.1	3	1.5	8	1.4
	Conserved feed	2.4	2.4	3.2	2.3	1.5	4.1	1.9	2.1	1.4	4	1.4
Housing	Kraal/Boma	66.5	42.5	91.8	40.3	83.4	33.4	33	28.2	44.5	34.8	12.4
	In house	4.4	6.2	7.1	4.4	5.5	10.6	20.6	20.6	15.4	11.2	71.4
	In stall with other animals	29.1	51.3	1.1	55.3	12.1	56	46. 4	51.2	41.1	54	16.2
Mating	Controlled	25.3	15.5	40.0	13.4	34.6	29.7	19.8	19.8	20.5	20.0	10.5
	Uncontrolled	74.7	84.5	60.0	86.6	65.4	70.3	80.2	80.2	79.5	80.0	89.5
Mating control methods	Castration	60.3	70.4	30.5	79.7	62.5	81.2	75.9	75.9	65.4	50.5	60.4
	Male separation	19.3	3.5	11.2	12.3	3.4	7.0	6.1	6.1	17.1	48.3	17.9
	Apron	20.4	12.9	58.3	8.0	34.1	11.8	8.0	8.0	7.5	1.2	8.5
Breeding management	Castration	20.4	12.9	36.3	16.8	56.3	25.2	44.1	41.4	17.9	26.3	13.8
	Culling	16.6	10.9	28.8	14.7	34.2	23.2	12.8	12.8	15.9	38.8	20.9
Culling criteria	Body condition	35.2	40.7	65.2	20.6	32.1	11.4	9.3	9.3	45.7	45.2	20.7
	Color	1.2	1.0	4.3	1.0	39.2	4.8	3.4	3.4	6	4.3	1.0
	Temperament	7.8	5.2	4.3	3.4	5.1	3.2	2.1	2.1	10.2	5.3	15.2
	Health	30.6	42.5	21.7	52.6	23.9	20.8	34.7	34.7	47.5	31.7	52.5
	Growth	15.2	8.4	26.1	5.4	20.2	23.3	18.2	18.2	13.4	30.1	8.4
	Old age	73.5	70.7	52.2	82.5	39.8	48.6	54.7	54.7	75.7	57.2	80.7
	Fertility	59.7	52.6	30.4	64.9	22.7	38.8	31.6	31.6	57.6	30.4	62.6

### Prevalent diseases and animal health management

Across all the study areas, the most prevalent diseases (Table 4) were gastrointestinal parasites (95.0%) and contagious caprine pleuropneumonia (CCPP), while cysticercosis was a relatively a big problem among the Pare goat keepers where more than 52% mentioned it. The majority (more than 66%) of the respondents reported having access to veterinary services from government extension officers and private veterinary practitioners. Common disease control measures employed by farmers was internal parasite control practiced by more than 68%. External parasite control was practiced by a majority of Maasai (92.2%), Pare (82%), and Tanga (55.5%) goat keepers while less than 45% of farmers who keep other goat types reported the practice. Across the study areas regular vaccination was done by less than half of the farmers for control of viral/bacterial diseases.

**Table 4:** Percentages of respondents for prevalent diseases of the goats and health management practices of the farmers in the study area

Disease/practice	Population										
	Gogo	Lindi	Maasai	Newala	Pare	Pwani	Fipa	Songwe	Sukuma	Tanga	Ujiji
CCPP	85.3	81	77	75.9	88.8	72.5	78.8	87.2	89	87	85.9
GIN	48.8	52.5	68.8	77.2	95.3	91	87	85.9	91	92.3	85.9
Cysticercosis	20.8	40.9	33.8	27.6	52.5	28.8	37.2	13.2	12.4	7.3	3.4
PPR	13.2	32.4	7.3	43.4	30.9	4.3	11	5	7.3	5.8	6.7
Mange	19.7	28.6	18.8	25.6	13.2	22.4	17.3	23.4	30.9	24.3	34.5
FMD	5.8	6.7	3.8	2.7	5	7.3	5.8	6.7	6.3	7.3	9.4
Anthrax	10.3	21.2	12.5	6.6	3.5	4.9	2.5	2.6	3.8	2.7	3.8
Anaplasmosis	7.3	9.4	6.3	2.4	8.5	2.3	7.5	7.5	2.5	7.3	9.4
Diarrhea	4.3	11	5	7.3	3.5	4.9	2.5	2.5	5	7.3	5.8
Footrot	3.5	4.9	2.5	2.5	7.3	9.4	6.3	2.4	3.8	2.7	5
Lumpy skin	8.5	2.3	7.5	7.5	5	7.3	5.8	11	5	7.3	5.8
Access to vet services	85.8	77.5	92.5	66.8	94.7	74.8	88.7	92.3	95.6	96.7	85.3
Regular vaccination	35.2	20.8	42.5	20.3	45.2	32.4	29.2	30.1	20.2	33.2	19.4
Internal parasite control	90	87.6	95	76.5	91.2	82	73.3	69.6	80.4	80.3	68.6
external parasite control	40	38.7	92.5	34.7	82	35	43.5	45.8	40.5	55.5	39

*CCPP* contagious caprine pleural pneumonia; *PPR* Peste des petits ruminants; *FMD* foot and mouth disease; *GIN* gastrointestinal nematodes

### Constraints associated with goat keeping

**Fig. 1:**
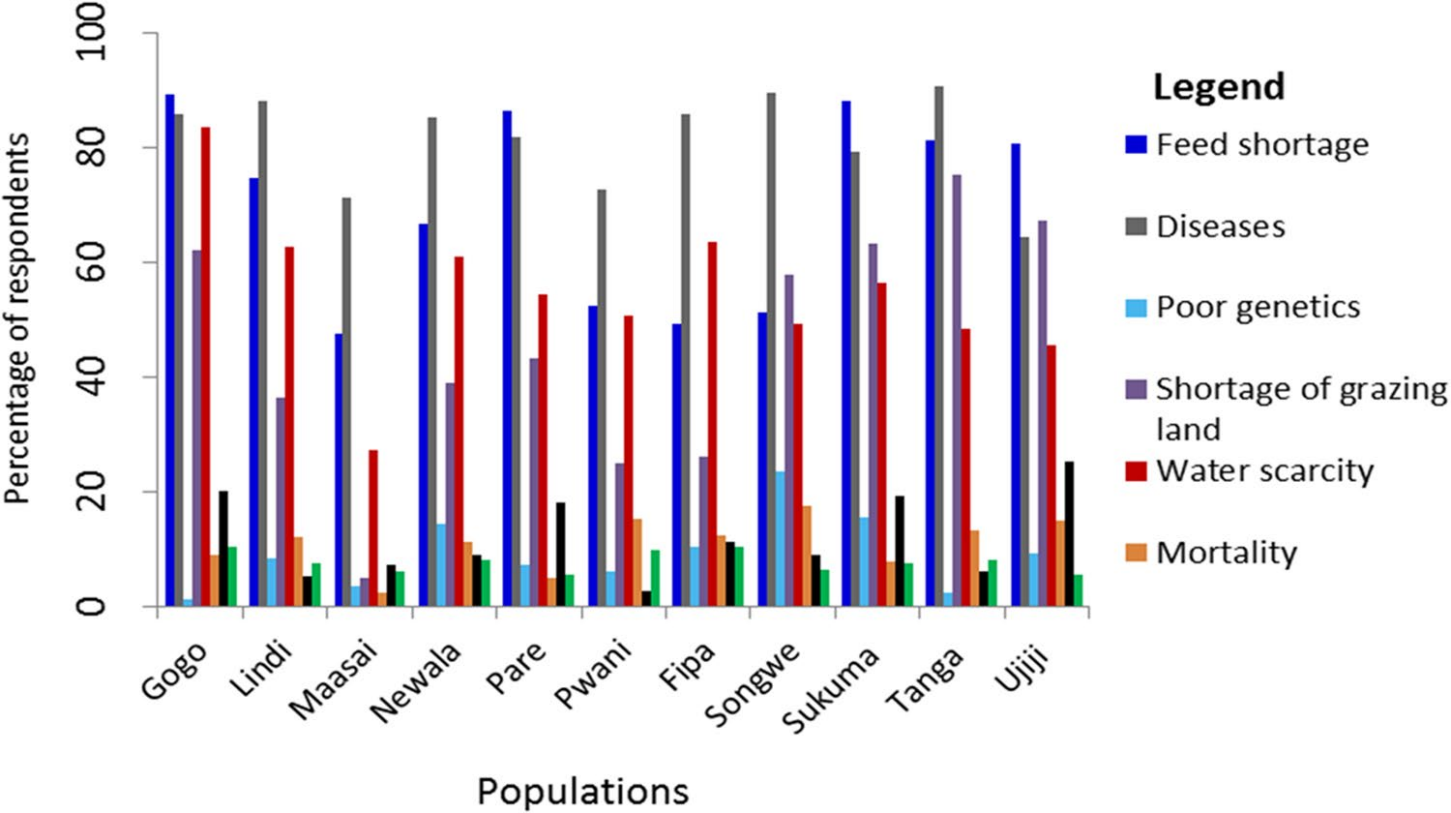
Major constraints to goat production in the study areas

Analysis of multiple response questions on the constraints to goat production by goat keepers in all areas is presented in Fig. 1. Results show that there were four main challenges: seasonal feed shortage (51–89%), disease prevalence (64–89%), water scarcity, and shortage of grazing land (25–75%). However, other challenges like mortality, livestock theft, poor genetics, and lack of extension services were mentioned but less frequently.

### Phenotypic description of Tanzanian indigenous goat populations

#### Qualitative characteristics

**Fig. 2:**
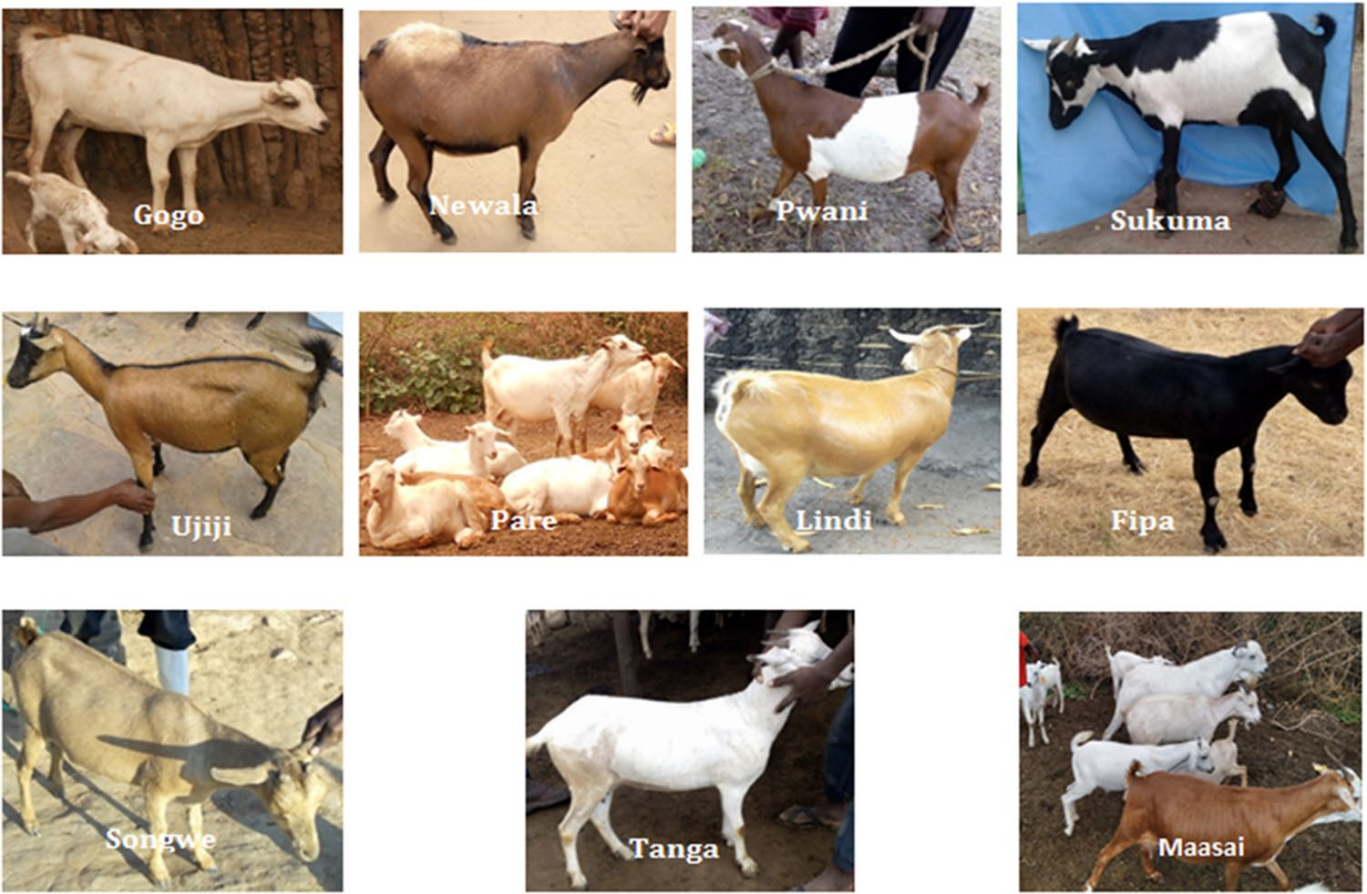
Physical variations of Tanzanian indigenous goat populations

The percentages of occurrence of qualitative traits of indigenous goats of Tanzania are presented in Table 5. The observed coat color patterns (Fig. 2) were plain and mixed whereby the percentage of goat population with plain pattern were 53.33% (Gogo), 50% (Lindi), 56.1% (Maasai), 87.04% (Newala), 69.12% (Pare), 33.33% (Pwani), 53.33% (Fipa), Songwe (58.33%), 25% (Sukuma), 58.33% (Tanga), and 44.29% (Ujiji). Furthermore, the majority of the goats with plain coat color were white (64.71%, 40.245, and 44.93% for Pare, Maasai, and Gogo, respectively) and brown for Newala (85.19%). The predominant mixed coat color patterns were black and white for Sukuma (50%), black and brown for Ujiji (45.71%), and white and brown for Pwani goat populations (44.4%). Other color patterns that were observed although in low frequencies were black and brown and black, white, and brown.

**Table 5:** Percentage of occurrence of different qualitative traits in indigenous goats in Tanzania

Variable	Category	Goat population										
		Gogo	Lindi	Maasai	Newala	Pare	Pwani	Fipa	Songwe	Sukuma	Tanga	Ujiji
Coat color type and patterns *	B	5.8	11.1	0	1.85	1.47	0	24.44	22.2	11.76	5.56	31.43
	W	44.93	1.85	40.24	0	64.71	25	0	2.78	2.94	33.33	2.86
	R-Br	2.9	38.89	15.85	25.19	2.94	8.33	28.89	27.78	8.82	19.44	10
	B and R- Br	8.7	12.96	0	63.7	1.47	0	4.4	8.33	10.29	2.78	45.71
	B and W	27.54	11.1	6.1	0	4.41	19.44	15.56	11.11	50	11.11	5.71
	B,W and R- Br	0.16	0	1.22	0	0	2.78	2.22	0	0	0	2.86
	W and R-Br	10.14	24.07	35.37	9.26	25	44.4	24.44	25	16.18	27.78	4.29
Wattle	Present	5.43	0	0	0	0	3.3	0	0	6.67	3.3	3.8
	Absent	94.57	100	100	100	100	96.7	100	100	93.33	96.7	96.2
Beard *	Present	45.65	0	9.1	13.6	54.17	6.7	0	0	33.33	0	3.8
	Absent	54.33	100	90.9	84.4	45.83	93.3	100	100	66.67	100	96.2
Ear size*	Large	20.65	0	0	0	2.08	0	0	0	10	0	0
	Medium	55.43	98	86.4	94	83.33	80	100	82.2	83.33	100	100
	Small	23.91	2	13.6	6	14.58	20	0	17.8	6.67	0	0
Ear orientation*	Horizontal	78.26	85.7	76.6	96	68.75	98	99	82.2	88.33	100	95.8
	Pendulous	15.22	0	3.3	0	31.25	0	0	0	0	0	0
	Erect	6.52	14.3	10.1	4	0	2	1	17.2	11.67	0	4.2
Horn	Present	96.74	96.4	89.1	99.8	97.92	70	100	96.4	100	90	93.6
	Absent	3.26	3.6	10.9	0.2	2.08	30	0	3.6	0	10	6.4
Horn shape*	Spiral	0	0	5.5	0	0	0	0	0	0	6.6	0
	Straight	67.39	43	63.2	78	47.92	36.7	25	40	83.33	38.1	38.5
	Curved	32.61	57	31.3	22	52.08	65.3	75	60	16.67	53.3	61.5
Horn orientation *	Upward	23.91	50	16.4	46.7	47.92	46.7	30	42.8	16.67	46.7	28.2
	Backward	73.91	50	83.6	53.3	52.08	53.3	70	57.2	80	53.3	71.8
	Lateral	2.17	0	0	0	0	0	0	0	3.33	0	0

*B* black; *W* white; *R-Br* reddish brown; *Br* brown. *Significant at *P* ≤ 0.05

#### Variation in quantitative traits among the Tanzanian indigenous goat populations

Results for body measurements of the different goat populations are presented in Table 6. Population-wise comparisons of least squares means of traits between populations revealed that Songwe and Tanga does were significantly (*p* < 0.05) the heaviest weighing above 32 kg and Sukuma goats the lowest weighing 24 kg, while other goat populations fell in between. Similarly, for other body dimensions, Songwe goats occupied the first position in body weight profile, while Sukuma occupied the lowest in all traits except for HW and HR in which Ujiji goats had the lowest value (Fig. 2). Generally, Songwe goats outperformed most of the other populations in all traits measured, while Sukuma and Ujiji had the lowest values in many of the traits measured. The CV for different traits ranged from 5.87% for HW to 17.29% for BW.

**Table 6:** Means and standard errors for body weight and body dimension for indigenous goat populations of Tanzania

Population	Variables				
	BW (kg)	BL (cm)	CG (cm)	HW (cm)	HR (cm)
Gogo	30.01 ± 0.68 cd	62.56 ± 0.56^bc^	73.10 ± 0.60^ab^	61.72 ± 0.46^ab^	64.38 ± 0.52^a^
Lindi	28.69 ± 0.82^de^	60.85 ± 0.67^ cd^	71.02 ± 0.72^ cd^	59.89 ± 0.56^ cd^	60.34 ± 0.63^de^
Maasai	30.69 ± 0.69 ^bc^	59.99 ± 0.56 ^d^	72.98 ± 0.60^ab^	62.31 ± 0.47^ab^	64.02 ± 0.53^ab^
Newala	29.64 ± 0.84 cd	63.72 ± 0.68 ^b^	69.17 ± 0.73^e^	58.59 ± 0.57^de^	62.12 ± 0.64^bc^
Pare	27.62 ± 0.71 ^e^	58.43 ± 0.58 ^e^	71.31 ± 0.62^ cd^	61.79 ± 0.49^ab^	64.17 ± 0.54^ab^
Pwani	28.69 ± 0.82 ^de^	62.78 ± 0.68^bc^	69.65 ± 0.72^de^	61.15 ± 0.56^bc^	62.94 ± 0.63^b^
Fipa	29.49 ± 0.83^cde^	59.52 ± 0.68^de^	71.58 ± 0.73^bc^	60.15 ± 0.57^c^	60.99 ± 0.64^ cd^
Songwe	32.74 ± 0.83 ^a^	66.51 ± 0.68 ^a^	74.30 ± 0.73^a^	61.58 ± 0.57^ab^	63.03 ± 0.63^ab^
Sukuma	24.06 ± 0.69 ^f^	55.88 ± 0.57 ^f^	67.34 ± 0.61^f^	58.03 ± 0.47^e^	60.51 ± 0.53^d^
Tanga	32.07 ± 0.83 ^ab^	60.68 ± 0.68 ^d^	71.10 ± 0.73^ cd^	62.65 ± 0.57^a^	63.82 ± 0.64^ab^
Ujiji	27.73 ± 0.74 ^e^	55.08 ± 0.61^f^	70.91 ± 0.65^ cd^	56.33 ± 0.51^f^	58.94 ± 0.57^e^
Overall mean	30.39	61.23	72.08	61.01	62.76
CV (%)	17.29	6.99	6.38	5.87	6.40

Means with different superscripts down the columns differ significantly (*P* ≤ 0.05)

*CV* coefficient of variation

*BW* body weight, *HG* heart girth, *WH* withers height, *BL* body length, *RH* rump height, *CD* chest depth, *EL* ear length, and *HL* horn length

#### Discriminant and population structure analysis

**Fig. 3:**
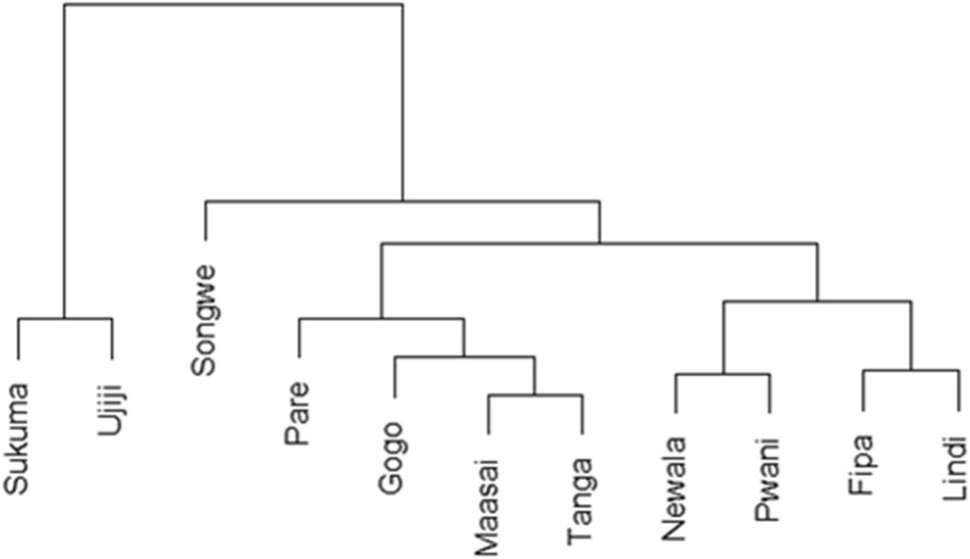
Dendrogram based on average linkage distance between adult goats using quantitative body measurement

The hierarchical cluster analysis generated a phylogenetic tree (Fig. 3) that clustered the 11 Tanzanian indigenous goat populations into two main groups that were not consistent with their geographical origins. The first group included only two goat populations (Sukuma and Ujiji), while the second group was made up of nine goat populations with further subdivision into three subgroups. Results for percent assignment of individual goats to their respective populations are presented in Table 7 below. On average, 36.4% of individuals were miss-assigned to her breeds. Ujiji, Newala, and Tanga goat populations had the highest proportion (more than 50%) of individuals correctly assigned to their source populations, while Pwani and Fipa had the lowest clarification rate (16.7%). While for most of the populations the misclassified individuals were found in all other populations, the misclassified individuals from Ujiji and Newala populations were missing in four populations, and Gogo and Songwe had their misclassified individuals missing in only one population.

**Table 7:** Percent of individual goats assigned to their respective populations

Source population	Correctly assigned (%)	Miss-assigned to other subpopulations (%)										
		Gogo	Lindi	Maasai	Newala	Pare	Pwani	Fipa	Songwe	Sukuma	Tanga	Ujiji
Gogo	24.7	-	4.1	6.9	1.4	7.8	11.0	0	19.2	4.1	4.1	6.9
Lindi	35.2	1.9	-	7.4	1.9	1.9	11.1	5.6	13.0	7.4	3.7	11.1
Maasai	22.0	6.1	2.4	-	1.2	22.0	1.2	3.7	1.2	13.4	20.7	6.1
Newala	55.6	3.7	0	0	-	1.9	13.0	0	11.1	7.4	7.4	0
Pare	48.5	5.9	2.9	10.3	1.5	-	1.5	2.9	1 1.5	13.2	8.8	2.9
Pwani	16.7	9.3	9.3	0	25.9	7.4	-	3.7	11.1	5.6	9.3	1.9
Fipa	16.7	3.7	16.7	5.6	7.4	5.6	3.7	-	5.6	9.3	9.3	16.7
Songwe	38.9	7.4	5.6	1.9	18.5	3.7	5.6	0	-	7.4	9.3	1.9
Sukuma	31.9	4.4	1.5	2.9	4.4	18.8	4.4	2.9	4.4	-	5.8	18.8
Tanga	51.90	1.9	3.7	1.9	7.4	3.7	9.3	1.9	3.7	0	-	14.8
Ujiji	57.8	0	0	7.0	4.2	0	0	1.4	4.2	23.9	1.4	-
Total	36.4	6.6	6.7	6.7	10.5	13.1	6.6	3.4	9.8	11.9	11.6	13.3

#### Canonical discriminant analysis

The Mahalanobis distances between the pairs of goat populations are presented in Table 8. The largest distance (7.58) was found between Ujiji and Songwe goats, while the closest distance (0.33) was recorded between Pare and Maasai goats.

**Table 8:** Squared Mahalanobis distance to pairs of population indigenous goats of Tanzania

From population	Gogo	Lindi	Maasai	Newala	Pare	Pwani	Fipa	Songwe	Sukuma	Tanga	Ujiji
Gogo	0										
Lindi	1.00	0									
Maasai	1.02	1.14	0								
Newala	2.86	2.88	4.77	0							
Pare	0.95	1.80	0.33	5.59	0						
Pwani	0.86	1.19	1.94	1.24	2.20	0					
Fipa	1.65	0.46	0.69	3.82	1.33	1.92	0				
Songwe	1.14	1.23	3.16	1.68	3.85	1.08	2.90	0			
Sukuma	2.44	1.93	1.74	4.43	1.52	2.52	0.93	4.84	0		
Tanga	2.66	2.18	1.09	3.96	2.21	2.03	1.61	3.92	3.18	0	
Ujiji	5.48	3.48	3.57	7.43	4.29	6.52	1.83	7.58	1.95	5.26	0

#### Reproductive performances

**Fig. 4:**
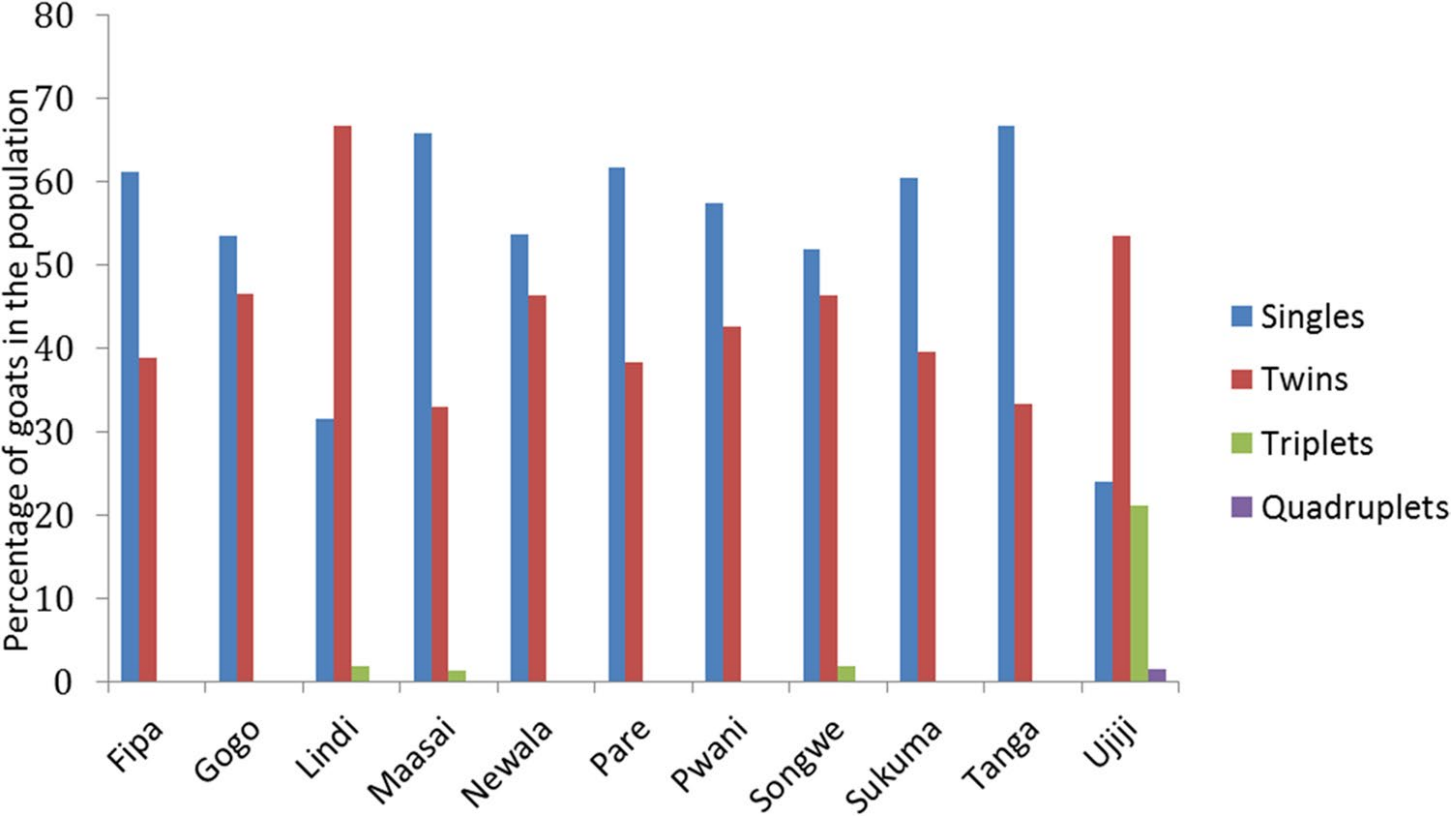
Occurrence of multiple births in Tanzanian indigenous goat populations

Incidence of multiple births and average number of kids at kidding which were the two parameters used to assess the reproductive performance of the goats is reported in Fig. 4 below. Incidence of multiple births was the highest among the Ujiji (76.07%) and Lindi goats (70.37%) and the lowest for Sukuma, Tanga, Maasai, Pare, and Fipa goats which had less than 40% of the animals giving birth to multiple kids at least once in their reproductive life. Ujiji and Lindi goats had an average of 2.0 and 1.7 kids in every kidding, while Pare had the smallest litter size at kidding of 1.09.

## Discussion

### Production objectives

This study is the first attempt to characterize the Tanzanian indigenous goats sampled from all major agro-ecological zones where they are raised and therefore representing all the presumed goat populations in the country. Previous efforts to characterize the indigenous goats in Tanzania have been concentrating on few populations or agro-ecological zones. For a sustainable breeding program, knowledge on purpose of keeping goats is essential (Van Arendonk 2011) because such knowledge is important in defining breeding goals and important features which affect motivation and profitability of long-term breeding programs (Jaitner et al. 2001). Results of this study revealed that goats in all the study areas are multi-functional and that financial functions were the most important in agreement with what was stated in earlier studies from other places in Africa (e.g. Nziku et al. 2016; Dossa et al. 2015; Berhanu et al. 2012). Farmers pay more attention on direct economic impact of the goats rather than their socio-cultural values. This was observed previously in Tanzania and was attributed to availability of markets and cultural changes as a result of modernization of rural communities (Nguluma et al 2016). Milk production was not given emphasis as a production objective of the farmers with exception of Pare goat keepers, and this can be attributed to cultural preferences for milk from cows since they co-exist with goats and lack of awareness of nutritional advantages of goat milk. Besides producing animal products including meat and milk, goats also provide manure to maintain soil fertility in mixed crop-livestock and agro-pastoral production systems and have socio-cultural roles to play. The purpose of the farmers of keeping goats have direct implication on their breeding goals which consequently affect their breeding strategies, therefore, they need to be given consideration in designing and running improvement and conservation programs. Integration with global market chains and abandoning of traditional livestock production systems results in shifting of the breeding goals to focus mainly on economic importance of the livestock. This has a consequence on conservation and continued existence of the valuable animal genetic resources including indigenous goats (FAO 2005).

### General goat husbandry practices

Management aspects including feeding, watering, and housing are determined by the production system which is influenced by the agro-ecological conditions of the particular area. Majority of the indigenous goats in Tanzania are raised under traditional extensive production system which is characterized by seasonal shortage of feeds and water. Additionally, majority of indigenous goat keepers are resource poor farmers who reside in marginal rural areas with limited supply of inputs where indigenous animals are adapted and therefore predominant. Under such production conditions, only the observed management practices of free grazing with minimal supplementation using locally available feed resources would be feasible. The findings in this study are consistent with study by Chenyambuga and Lekule (2014) who reported heavy reliance upon natural pastures available in communal grazing lands for feeding goats with little supplementary feeding using maize bran, Sorghum, and maize stovers during the dry season after crop harvest.

It can be noted that in areas like Mtwara, Pwani, Tanga, Rukwa, and Songwe where mixed crop livestock is the dominant production system, the proportion of farmers who practiced tethering along with free grazing was relatively higher. This was due to shortage of grazing lands especially during wet season when most of the land is taken for growing crops leaving no or little land available for heading and free grazing. Also, it is common during the rainy season for most of the family members to be working on their farm plots and therefore farmers with few animals tether their animals close to their farm plots or their homesteads or leave them indoors and bring them feed when returning from field work.

The observed housing systems across the study areas do not offer protection of animals against predation, theft, and weather extremes which could lead to low productivity of the animals. Poor housing of animals has been observed in many smallholder systems in the tropics (Gwaze et al, 2009) and is attributed to lack of knowledge and financial resources of the farmers (Shumba, 1993). Therefore, awareness creation among goat farmers on the importance of improved housing in the performance of goats is important. Low production potential which is usually associated with indigenous goats in the tropics is confounded with low standard of management under which the goats are normally kept (Mpofu, 2002). Therefore, in order for indigenous goats in Tanzania to perform to the expectation and requirement of the farmers, any genetic improvement strategies must be accompanied with improvement in the management of the animals ((Philipsson et al. 2006).

### Breeding practices

Controlled mating is one of the best livestock breeding management practice for improvement of animal productivity to be achieved. Controlled mating is important for genetic improvement as it enables farmers to avoid indiscriminate crossbreeding and inbreeding. Also controlled mating enables farmers to plan for their animals to kid at a time when there is sufficient amount of feeds typically after the rain season. Low level of mating control is caused by several factors including poor land tenure system in which individual land ownership is not encouraged and communal grazing and sharing of watering points is common, typical of extensive production system which is the most predominant in Tanzania. Castration, though not very common in most parts, was the most widely used method among the farmers who reported to practice controlled mating. Apart from controlled mating, improving the quality of meat through fattening and reducing bad smell from bucks to get better price in markets were another motivation for castration. Separation of bucks could work better in controlling mating, but under small-scale farming systems, this could be hard and expensive to have few separate groups of bucks. Considering the low practice of castration and culling against old animals especially bucks, the use of breeding bucks from within the flock as observed among the interviewed farmers represents a high risk of inbreeding of which they were probably not aware.

### Health management and disease control

In order to develop sustainable strategies for control of small ruminant diseases, there is a need to determine the most important diseases affecting the animals in different areas (Shija et al 2014). The major goat diseases of helminthosis, CPPP, and PPR reported by the farmers across the study areas have been mentioned previously by other researchers (Chenyambuga and Lekule 2014; Nguluma et al 2020; Onditi et al. 2007; Mbyuzi et al. 2015; Shija et al 2014). Nguluma et al. (2020) reported high incidences of helminthosis and CCPP among the Maasai goats and associated the prevalence to management systems of the farmers. High contamination of pastures with eggs or larvae leads to high incidences of helminthosis and overcrowding in watering points, and grazing and poor housing expose animals to stressful weather conditions and increase the chances for CCPP to spread between and within herds. High cases of CCPP and PPR have been reported in southern regions of Tanzania, native to Newala and Mtwara goats, and the increase in cases has been associated with purchase of animals from outside to replenish stocks following increased slaughter of goats during the time of festivals commonly January to February of every year. The practice brings in animals from other goat rearing zones which are already infected and endemic for CCPP and PPR. Most of the disease challenges reported by the farmers result from poor management, including poor housing, inadequate feeding and feeding systems, irregular disease control strategies, thus improvement in management will likely alleviate the problem. In addition to providing knowledge and awareness to farmers on the importance of management on disease occurrence, knowledge on importance of adhering to proper veterinary drug use will limit recurrence of diseases and cut drug resistance that might arise from their improper use. In a previous study in one of the study sites, Nguluma et al. (2020) noted excessive use of veterinary drugs from unauthorized dealers and treatment of animals by farmers without consulting authorized veterinary officers despite the availability and easy access to veterinary services from qualified veterinary practitioners.

### Constraints to goat production

Goat production in communal areas is faced with many constraints which may differ with areas, countries, regions, or geographical locations (Kosgey 2004). The major constraints facing goat farmers in the study areas differed, but the major ones were similar across the study areas. Similar to the observation in this study, high prevalence of diseases and parasites and feed and water shortage as well as drought have been reported by other researchers in Tanzania (Nguluma et al., 2020; and elsewhere in Africa as most influencing constraints to goat production (Raghuvansi et al. 2007; Ben Salem and Smith 2008; Gatew 2014)). Contrary to the findings in this study, Chenyambuga and Lekule (2014) reported animal health problems not to be the major concern of the goat keepers in central Tanzania attributing this to tolerance of indigenous goats to endemic diseases.

### Variation in quantitative traits among the Tanzanian indigenous goat populations

Description of the animal phenotypic features in terms of body measurements is important in making taxonomic, behavioral, and ecological comparisons within and between animal populations and explaining intraspecific variation in morphology over broad environmental gradients (Mittelbach et al., 2007). Body weight is a trait of economic importance in livestock production and one of the most preferred traits reflecting farmers’ main production objectives of income generation and meat production. The ranges of 24 to 32 kg of body weight of indigenous goats in the study areas were above the range of 20 to 25 kg that has been reported previously for other mature SEA goats in traditional farming (NEI 1999). Similar to the findings of this study, Madubi et al. (2000) reported body weight of 31.8, 29.2, and 23.9 kg for Gogo, Newala, and Ujiji goats, while Nguluma et al. (2016) found Pare and Sukuma to weigh 29.8 and 22.3 kg, respectively. Body weight of Sukuma and Ujiji which were the smallest of the goat populations in the present study was above the average weight of 14.51 kg reported for dwarf goats of West Africa (Rotimi et al. 2015). However, the body weights of the indigenous goats in the study areas are lower than that of Blended goats, a famous composite breed kept for meat in Tanzania, which was reported to weigh above 40 kg (Das and Sendalo 1991).

Variations observed in body weight and body dimension among the indigenous goat populations may be due to isolation-by-distance, historical and geological factors, physical barriers, and ecological factors through morphological adaptation to local conditions (Mekuriaw et al., 2016). The indigenous goat populations studied are found in different geographical areas with varying ecological characteristics. Ecological variations influence the body measurements of the goats through differences in feed and water availability and environmental temperature. Based on wither height measurements, Devendra and Burns (1983) classified goats as large if they were above 65 cm, small to medium if they measured between 51 and 65 cm, and dwarf for those with wither height below 50 cm. Based on this classification, indigenous goats in Tanzania can be categorized as small- to medium-sized. Phenotypic features are influenced by the environment as well as genetic constitution of the animal; therefore, it is difficult to conclusively associate body measurements to any genetic background or the ecological variations of study areas. Traits like height at withers and body length have been reported to be more genetically determined, while heart girth is more subject to environmental influences (Searle et al. 1989; Hall 1991). The CV obtained in this study for quantitative traits was low (6.87–17.29) which indicates small differences among populations for the traits studied and lack of selection in the different populations for the traits. Studies of variation of the goats at molecular level may reveal the genetic basis of the variation which is important if higher accuracy of selection is to be achieved since in breeding programs it is the heritable part of the variation that can bring about the desired genetic improvement through selection.

### Variation in qualitative traits among the Tanzanian indigenous goat populations

Significant variations were observed in terms of qualitative traits of the goats across the study areas. In the present study, the common colors were plain white, black, and reddish brown and mixture of black and white, black and reddish brown, white and reddish brown and black, and white and reddish brown. The findings are consistent with observations of Mason and Maule (1960), who reported the common colors of indigenous goats in Tanzania to be black, brown, white, and gray occurring in various combinations of bi-color or multi-color. However, some goat populations could clearly be distinguished by a predominant color or color combinations. For example, Gogo, Maasai, and Pare goats were predominantly white colored though combinations of white and other colors were observed in lower frequencies. Majority of Newala were plain reddish brown, while Sukuma had a mixture of black and white. Qualitative traits do not have a direct economic importance but have socio-cultural values to the communities; therefore, some farmers have specific preference for some traits (Mahanjana and Cronje 2000; Gwaze et al. 2009). Due to this specific preference, frequency of some traits may be higher in the population due to unintentional selection for these traits for certain socio-cultural roles that the goats play. White-colored goats are preferred during traditional rituals or offering of spiritual sacrifices among Pare goat keepers which motivates selection and maintaining of white-colored breeding animals. Similarly, Mahanjana and Cronje (2000) reported white goats to be in high demand for sacrificial purposes, and comparatively high prices were paid for them in the Eastern Cape, South Africa. Selection for qualitative traits may automatically have an impact on quantitative traits due to genetic correlation that exists between them (Yakubu et al., 2010). Additionally, direct selection pressure exerted on animals due to differences in ecological and climatic conditions of the study areas may affect their presence and appearance as adaptive mechanisms. For example, coat color type and patterns and the presence or absence of wattle play a significant role in temperature regulation and, therefore, adaptability of the animals to the environment. Consequently, alleles controlling these features may be favored by natural selection causing their frequency to increase in that population (Yakubu et al., 2010). Other reasons given for preference of certain colors in indigenous goats in Tanzania include security of animals during grazing (Nguluma et al, 2016) and adaptation to climatic conditions (Msemwa and Mbaga, 2018). Therefore, inter-population comparison for qualitative characteristics may result from differences in eco-geographical and sociological conditions of the areas where the goats are found.

### Multivariate analysis

High percentages of correctly assigned individuals for Ujiji, Tanga, and Newala goats are an indication of more uniformity and homogeneity of these populations which might have been caused by reproductive isolation and good production conditions. Ujiji goats are found in Kigoma region which is located in the north-western part of the country bordering DRC and historically has not experienced as much interaction with pastoralists from other regions compared with many other parts of the country due to its peripheral location. Similarly, Newala goats native to Mtwara region are located in the southern-eastern border with Mozambique which until recently had limited movement of pastoralists from other parts of the country into the region due to prevalence of cattle trypanosomosis. Tanga goats from Tanga region found in the Eastern part of the country close to the Indian Ocean has sub-humid weather and with good rainfall which affect the quality and quantity of pasture forage.

On the contrary, the low classification rate of Gogo, Maasai, Pwani, and Fipa goats indicates the heterogeneity of the population due to intermixing with different populations because of geographical closeness and interactions between goat keepers or similarity of production conditions. Consistent with the discriminant analysis, canonical discriminant analysis revealed that inter-population distance was small and insignificant for most of the pairs of goat populations reflecting their geographical distance and possibility of intermixing due to pastoral migrations. Even where the goat populations are not geographically close, like the case of Maasai and Fipa, the populations might be in the same “migration route” which bring the animals in contact. Pastoral and agro-pastoral communities like Sukuma, Maasai, Barabaig, Kurya, and Taturu were reported to be migrating with livestock in different parts of the country (Tenga et al, 2008). The Sukuma community migrated from the Lake Zone to the Lake Rukwa basin which is home to Fipa goats and later to Usangu and Morogoro plains close to where Pwani goats are found (ibid). The Maasai from the northern Tanzania migrated to Morogoro and Usangu plains before independence (Lukumbo 1998; Walsh 2007). Implementation of programs for improvement of productivity under such situations where farmers are in constant movement and unplanned mixing of goat genotypes is difficult and unlikely to achieve any significant impact. Furthermore, due to this haphazard intermixing of animals from different agro ecologies, the diversity and unique genetic features possessed by different indigenous goat genotypes cannot be properly utilized for improvement in productivity. A study by Tenga et al. (2008) recommended a better organized, consistent, and more broad-based approach in the area of policy advocacy, legal issues, and investment issues for efforts to secure the animal resources that have sustained pastoralists in the past to have an impact.

### Reproductive performance of the indigenous goats

Animals producing twins or triplet contribute more than 1.5 times towards meat production than the animals producing single offspring at birth (Khosa, 2012). Twinning ability has been reported to be one of the most preffered traits by goat keepers in Tanzania (Nziku et al. 2016), and it reflects the economic efficiency in meat goat industry. The average litter size of 1.82 for Ujiji goats in this study is quite comparable with some world prolific goat breeds including Nubian, Pygmy, American Alpine, French Alpine, Saanen, and Toggenburg with the average litter size of 2.0, 1.9, 1.9, 1.7, 1.7, and 1.6, respectively (Amoah et al., 1996), suggesting that Ujiji goats are a prolific goat breed. Twining ability has low heritability (0.07 for triplets and 0.02 for twins) (Cottle et al. 2016) and is influenced by management. For example, Cottle et al. (2016) reported a high-energy diet to be beassociated with a greater proportion of multiple births. Variation between Ujiji goats, the most prolific population, and Sukuma goats the least prolific was about 46% implying that crossbreeding between different goat populations would increase the prolificacy in the indigenous goats. However, increasing litter size is known to reduce birth weight of kids (Amoah et al., 1996) consequently affecting their preweaning survival. Therefore, breeding for high twinning ability should be accompanied with good management of does to bring them up to a reasonable good mating weight or condition to improve litter size while providing good-sized offspring.

## Conclusions

Generally, the indigenous goats in Tanzania are heterogeneous and have very small between population variations and large within population variations. The 11 goat populations can be clustered into two major groups based on body weight and body dimensions with Ujiji and Sukuma occupying one cluster and other cluster comprising of all other populations with minor clusters between them. Notable distinction has been observed in terms of twinning with Ujiji and Lindi goats showing high ability. The large variation observed especially within populations, with regard to body weight and body dimension, and between populations with regard to reproductive performance, is important as it can be used as a basis for genetic improvement through selection and/or crossbreeding.

## Data Availability

The datasets generated during and/or analyzed during the current study are available from the corresponding author on reasonable request.
